# Book Reviews

**Published:** 1895-05

**Authors:** 


### Book Reviews.

---

TWENTIETH CENTURY PRACTICE OF MEDICINE—An International Encyclopedia of Modern Medical Science, by leading authorities of Europe and America, edited by Thomas L. Stedman, M. D. New York City. In twenty volumes. Vol. I., Diseases of the Uropoietic System. New York: William Wood & Company. 1895.

We have received the first volume of this exceedingly ambitious work, and we think the effort of the editor to give to the profession a work of this kind is a laudable one, and we extend to him our hearty congratulations, for we feel that if Vol. I. is a foretaste of what is to follow, his success is already assured.

To the specialist in diseases of the uropoetic system, Vol. I. is of untold value and the general practitioner cannot afford to be without it.

The first article on diseases of the kidneys by Dr. Francis Delafield, of New York, and the second on diseases of the kidneys (surgical) and of the ureters, by Dr. Reginal Harrison, London, are very ably handled and should be carefully studied by every practitioner.

The third article is on "Diseases of the Bladder" and is by Dr. Reginald Harrison also; it is well-written and very instructive.

The fourth and fifth articles, "Diseases of the Prostate" and "Diseases of the Male Urethra," are by Dr. G. Frank Lydston, Chic go. Too much cannot be said of the clear and practical manner in which they are written, and of the great amount of information they contain.

Following these is a very readable article on "Diseases of the Urine," by Dr. E. Harry Fenwick, London. Professor Howard A. Kelly, Baltimore, contributes the article on "Diseases of the Female Bladder and Urethra," with which the volume closes. We were agreeably impressed with this article because of its exceedingly practical character.

---

ANTISEPSIS AND ANTISEPTICS—By C. M. Buchanan, M, D., Professor of Chemistry, Toxicology, National University, Washington, D. C., with introduction by Prof. Augustus C- Bernays, of St. Louis; Published by the Ferhune Company, Newark, N. J.

This little work will be truly appreciated by the busy practitioner on account of its condensed form. The convenience of finding at a glance the subject treated, or the authors quoted, can be readily understood. The details and rules, if followed will accomplish much good. Price, $1.75.

INTERNATIONAL CLINICS: A QUARTERLY OF CLINICAL LECTURES ON MEDICINE, NEUROLOGY, PEDIATRICS, SURGERY, GENITO-URINARY, GYNECOLOGY, OBSTETRICS, OPHTHALMOLOGY, LARYNGOLOGY, OTOLOGY AND DERMATOLOGY. BY PROFESSORS AND LECTURERS IN THE LEADING MEDICAL COLLEGES OF THE UNITED STATES, FRANCE, GREAT BRITAIN AND CANADA. Edited by Judson Daland, M. D., Philadelphia; J. Mitchell Bruce, M. D., F. R., C. P., London, Eng., and David W. Finlay, M. D., F. R., C. P., Aberdeen, Scotland. Vol. 2, 4th series. J. B. Lippincott Company, Philadelphia, Publishers, 1895.

This volume is of unusual interest, and should be in the hand of every practitioner who wishes to keep abreast of the times. Some of the subjects treated are probably of more interest to the general practitioner than others, but the matter throughout is practical, well-arranged and of great value.

---

A book of unique character which will undoubtedly attract more than ordinary attention is announced for early publication by S. C. Griggs & Co., Chicago. Under the title "Dr. Judas," the author, Mr. William Rosser Cobbe, a well-known Chicago journalist, has depicted with an unusually facile pen and with rare descriptive powers, the terrible experience of an "opium fiend" of nine years standing. He writes from personal experience with what he aptly terms the Judas of drugs; describing, in all its phases, the mental, moral, and physical degradation of the victim, stripping the habit of its glamour and deceit, and revealing to the uninitiated the disastrous consequences of indulgence in this most dangerous of addictions. Due attention is also given to other toxic agents, as cigarettes, chloral, cocaine, hasheesh, etc.

In this work the author seeks to enlighten the public respecting opium, to warn of the dangers of its employment by physicians, to enlist co-operation in effort to regulate its sale and to assure all addicted to its use of the certainty of a complete release from its bonds.

To the general reader this book will be of interest because of its fascinating literary style, while it will prove of incalculable value to the physician because of its graphic portrayal of the effects of opium upon the body; to the lawyer for the important bearing of its facts upon jurisprudence, and to the teacher as a guide in warning the young against the baneful influences of all forms of pernicious and enslaving drugs.

